# Predictors of chest wall toxicity after stereotactic ablative radiotherapy using real-time tumor tracking for lung tumors

**DOI:** 10.1186/s13014-017-0803-2

**Published:** 2017-04-05

**Authors:** Younghee Park, Hee Jung Kim, Ah Ram Chang

**Affiliations:** Department of Radiation Oncology/CyberKnife Center, Soonchunhyang University Seoul Hospital, 59 Daesagwan-ro, Yongsan-gu, Seoul, 140-743 Republic of Korea

**Keywords:** Stereotactic ablative radiotherapy, Lung tumor, Chest wall toxicity, Rib fracture, Dose-volume histogram, 4D dose calculation

## Abstract

**Background:**

To evaluate the incidence of chest wall toxicity after lung stereotactic ablative radiotherapy (SABR) and identify risk factors for the development of rib fracture.

**Methods:**

Thirty-nine patients with 49 lesions underwent SABR for primary or metastatic lung tumors using Cyberknife® with tumor tracking systems. Patient characteristics, treatment factors and variables obtained from dose-volume histograms (DVHs) were analyzed to find the association with chest wall toxicity. Four-dimensional (4D) dose calculations were done to investigate the effect of respiratory motion on dose to the ribs.

**Results:**

After follow-up of median 26.7 months (range: 8.4 – 80.0), 8 patients (20.5%) experienced rib fractures and among these patients, three (37.5%) had chest wall pain at 2–3 months after SABR. Median time to rib fracture was 13.4 months (range: 8.0 – 38.5) and the 2-year actuarial risk of rib fracture was 12.2%. Dose to the 4.6 cc of the ribs (D_4.6cc_) and rib volume received 160 Gy or more (V_160_) were significant predictor for rib fracture. No significant differences between three-dimensional (3D) and 4D dose calculations were found.

**Conclusions:**

Parameters from DVH are useful in predicting the risk of chest wall toxicity after SABR for lung tumors. Efforts should be made to reduce the risk of the rib fracture after lung SABR.

## Background

Excellent local control rates have been reported in stereotactic ablative body radiotherapy (SABR) for early stage lung cancer [1–3]. Based on the promising treatment outcome equivalent to surgery, SABR is considered as an alternative treatment not only in medically inoperable patients, but also in operable patients [4–6]. Higher dose to the tumor improves local control rate, but at the same time, it also increase the dose to the adjacent normal tissues. In general, SABR is well tolerated and the reported incidences of acute and late toxicities are low. However, use of large dose per fraction and increased survival of patients raised concerns about late toxicities different from conventional radiotherapy. Several studies reported the chest wall toxicity such as radiation induced rib fracture (RIRF) or chest wall pain as late toxicities of SABR. The reported incidences of chest wall toxicity in SABR are generally higher than those in conventional radiotherapy and vary widely among studies [7]. Chest wall or rib is considered as an important organ at risk in lung SABR, but consensus on dose constraint for these has not been reached [8–10].

Cyberknife® is an image-guided radiotherapy system with linear accelerator mounted on a robotic arm [11]. It provides a real-time tumor tracking system which corrects for respiratory motions by moving robotic arm [12]. Therefore, using tumor tracking systems, it is expected to provide more accurate targeting of tumors and lower radiation dose to adjacent normal tissues. Previous studies demonstrated the efficacy of SABR using Cyberknife® and real-time tumor tracking in early stage lung cancer [13–15]. A recent study by Roth et al. reported that dosimetric and radiobiologic indices were comparable between Cyberknife® and LINAC-based SABR, however, the comparability was not verified in terms of clinical outcome or toxicity [16].

The purpose of this study is to report the incidence of chest wall complication in patients with primary or metastatic lung tumors treated with SABR using real-time tumor tracking and to identify adequate dose constraints for ribs by evaluating the dose-volume parameters.

## Methods

### Patients

We retrospectively evaluated total 44 patients who ﻿were treated with SABR for primary or metastatic lung tumors from July 2008 to October 2014 at Soonchunhyang University Seoul Hospital. Patients with follow-up duration less than 6 months were excluded in this study. Multiple lesions were considered as separate if their dose distributions were minimally overlapped (no overlapped ribs between isodose lines of 10 Gy in 2 Gy equivalent dose, EQD2). In case of re-irradiation or adjacent lesions of previous radiotherapy, the lesions were regarded as one lesion and dose distributions of each plan were summated on fused computed tomography (CT) images. Final 39 patients with 49 lesions were analyzed. Ten patients with 20 lesions received multiple course of thoracic radiotherapy including both conventional radiotherapy and/or SABR. Four patients with 11 lesions received different course of SABR to separate lesions in which their dose distributions were not overlapped to each other. One patient received SABR to the recurrent lesion of previous SABR and one to the recurrent lesion of previous conventional radiotherapy. Two patients had two adjacent lesions all treated with SABR so those lesions were considered as one, and summated dose distributions were analyzed for dosimetric evaluations.

### Radiotherapy

All patients were treated with Cyberknife® (Accuray, Inc., Sunnyvale, CA, USA) and immobilized with Vac-Lok® (MEDTEC, Orange City, IA, USA) in supine position. Treatments were delivered with real-time tumor tracking using X-sight® Lung (Accuray, Inc., Sunnyvale, CA, USA) or fiducial-based target tracking (Synchrony® (Accuray, Inc., Sunnyvale, CA, USA). A fiducial marker was inserted at the center of tumor in 35 lesions (71.4%). Gross tumor volume (GTV) was contoured on lung window setting and planning target volume (PTV) was generated by adding 3–5 mm margin to the GTV. Total dose of 45–66 Gy was prescribed to the 80% isodose line in 3 to 6 fractions depending on the risks of surrounding normal organs. Dose to the ribs were not constrained on the original treatment plans.

### Clinical evaluation

All the medical records of included patients were retrospectively reviewed and any symptoms related to chest wall complication were assessed. Follow-up CTs on the bone window setting were reviewed to detect the rib fracture and the time interval between the first day of SABR and initial appearance of rib fracture was measured. Only newly detected rib fractures within the SABR field were recorded as RIRF and fractures secondary to tumor recurrence or progression were excluded. Rib fracture and chest wall pains were graded according to the criteria for “fracture” and “pain” in Common Terminology Criteria for Adverse Events (CTCAE, v4.0). Chest wall pain existed before SABR with no change in its intensity after SABR or those not correlated with the treatment field were not considered as radiation induced toxicity.

### Dosimetric evaluations

Because patients were treated with various dose fractionation schedules, we calculated the EQD2 with α/β ratio of 3 Gy for ribs [17–19]. All ribs receiving EQD2 of 10 Gy or more were contoured on the planning CT and dose-volume histograms (DVHs) were generated. Based on the DVH, the maximum point dose of irradiated rib (D_max_), absolute dose received by rib volumes of V (D_V_) and absolute rib volume receiving a threshold dose D (V_D_) were calculated.

### 4D dose calculations

We have used 4-dimensional (4D) CT scanning technique since 2013 to monitor the tumor motion and to use for contouring and evaluating the target coverage with respiration although only 3-dimensional (3D) dose calculations have been used for all clinical treatment planning. Dose calculations of these patients were done using Monte Carlo algorithm. To evaluate the accuracy of 3D dose calculation for ribs, full sets of 4D CT images for 9 lesions from 7 patients were successfully reloaded. The raw data of 4D CT were binned into 10 phases and ten 3D CT datasets were reconstructed. Each respiratory phase was registered based on fiducial markers using MIM software (MIM Software Inc. Cleveland, OH). After the image registration, the cumulative dose was determined by combining dose distribution of each phase.

### Statistical analysis

All statistical analyses were performed using SPSS version 18.0 (SPSS Inc., Chicago, IL). Actuarial rates of rib fracture, local control and overall survival were calculated with Kaplan-Meier Methods and the log-rank test was used to compare the risk factors on univariate analysis. Cox proportional-hazard model was used for multivariate analysis. The receiver operating characteristic (ROC) curves were generated to assess the optimal cut-off values of DHV parameters and their predictability of rib fractures. Areas under the curves (AUCs) were calculated to compare the cut-off values. *P*-values less than 0.05 were considered to be statistically significant.

## Results

The median follow-up was 26.7 months (range, 8.4 – 80.0 months). The local control rates at 2 years were 88.0%. The patients and tumor characteristics are summarized in Table 1. Median age at SABR was 66 years (range, 46 – 84 years). More than half of the patients (57.1%) had primary or recurrent lung cancer lesions and 30 lesions (61.2%) were located in middle or lower lobe. Median GTV volume was 5.90 cc (range, 0.79 – 111.74 cc) and median distance between rib and tumor was 0.25 cm (range, 0.00 – 4.10 cm).

**Table 1:** Patients and tumor characteristics

Characteristic		Number (% or range)
Age (year)	Median	66 (46–84)
Sex	Male	23 (59.0)
	Female	16 (41.0)
Primary site	Lung	28 (57.1)
	Breast	3 (6.1)
	Colorectal	16 (32.7)
	Others	2 (4.1)
DM	Yes	11 (22.4)
	No	38 (77.6)
COPD	Yes	9 (18.4)
	No	40 (81.6)
Tumor location	Upper lobe	19 (38.8)
	Middle and lower lobe	30 (61.2)
GTV volume (cc)	Median	5.90 (0.79 – 111.74)
Rib-tumor distance (cm)	Median	0.25 (0.00 – 4.10)

*DM* diabetes mellitus, *COPD* chronic obstructive pulmonary disease, *GTV* gross tumor volume

### Chest wall toxicity

**Fig. 1:**
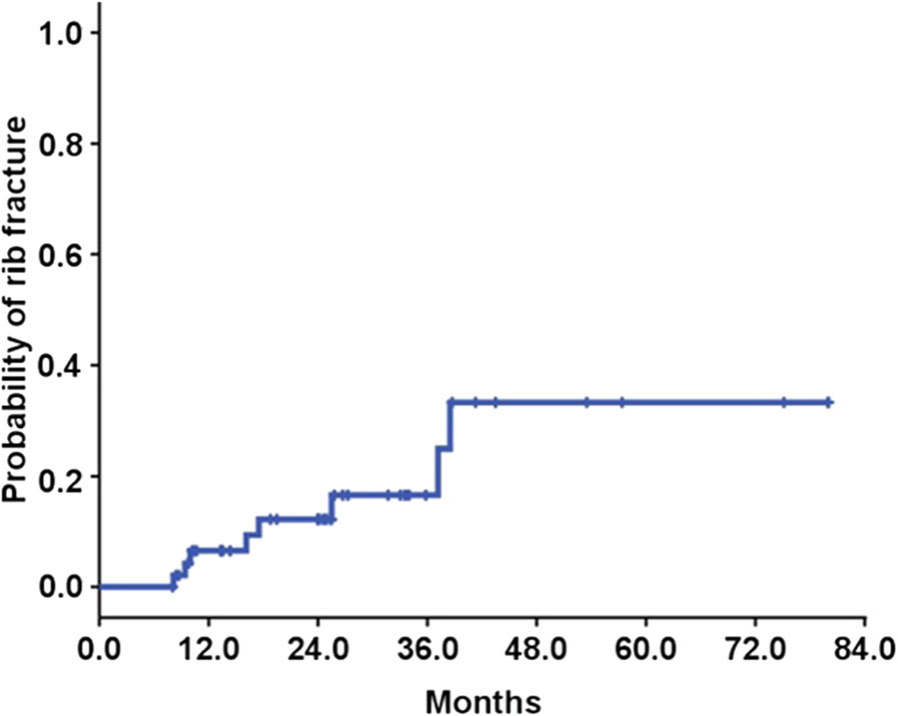
Cumulative incidence of rib fracture after SABR

Rib fractures were identified in 8 patients and actuarial incidence of rib fracture was 12.2% at 2 years and 16.6% at 3 years (Fig. 1). Five patients had Grade 1 rib fractures, one had Grade 2 and two had Grade 3. Time interval from SABR to the rib fracture was median 13.4 months (range, 8.0 – 38.5 months). Three patients complained of Grade 1 chest wall pain at 2–3 months after SABR without any evidence of rib fracture at that time. One patient need non-opioid analgesics and the other two did not need any analgesics. All these patients eventually developed Grade 3 rib fractures after longer follow-up, but the statistically significant associations were not found between chest wall pain and Grade of rib fractures. There was no patient who had chest wall pain alone without rib fractures. Therefore, we analyzed the risk factor for rib fractures in subsequent analysis.

### Dose-volume analysis of rib fracture

From DVHs, D_max_ and D_0.1cc_ to D_10cc_ were derived in 0.1 cc increments from 0.1 to 5 cc and 1 cc increments from 5 cc to 10 cc. Volume of ribs receiving EQD2 of 50 Gy to 300 Gy were also obtained in 10 Gy increments. The AUCs of each parameter were calculated and the maximum AUC of D_V_ was at 4.6 cc (*p =* 0.010) and V_D_ was at EQD2 of 160 Gy (*p =* 0.005), and these parameters were selected as predictors for rib fractures. Based on the sensitivity and specificity, 140 Gy and 3.2 cc volume of rib were selected as cut-off values for D_4.6cc_ and V_160_, respectively.

### Clinical and dosimetric risk factors for rib fracture

**Fig. 2:**
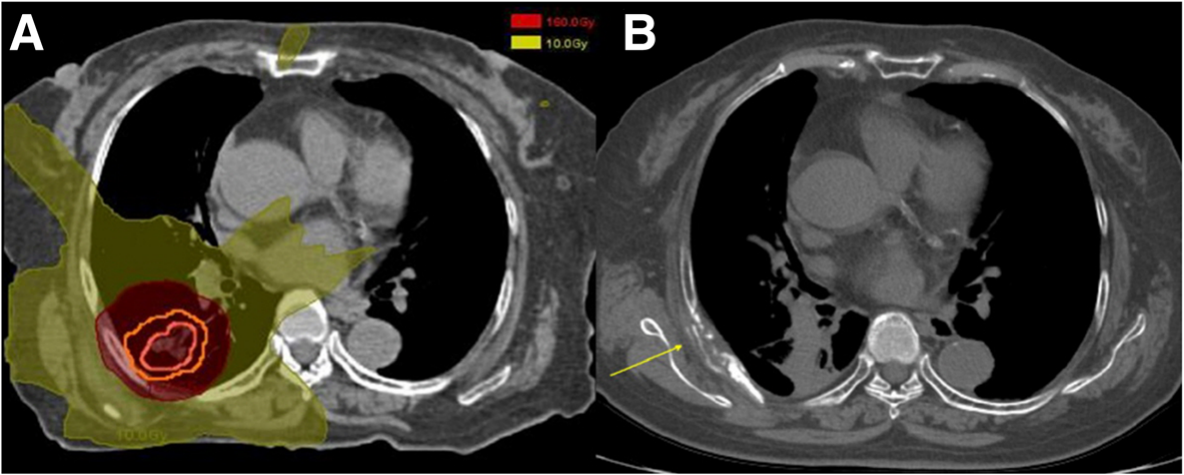
Example of radiation induced rib fracture and dose distribution. A 74-year-old man treated for adenocarcinoma in *right lower lobe* developed rib fracture after 37 months. The prescribed dose was 66 Gy in 3 fractions. **a** Dose distribution in the transverse plane corresponding to the site of rib fracture. *Red area* represents the *isodose line* of 160 Gy in EQD2. **b** Rib fracture detected on follow-up CT (*arrow*)

**Fig. 3:**
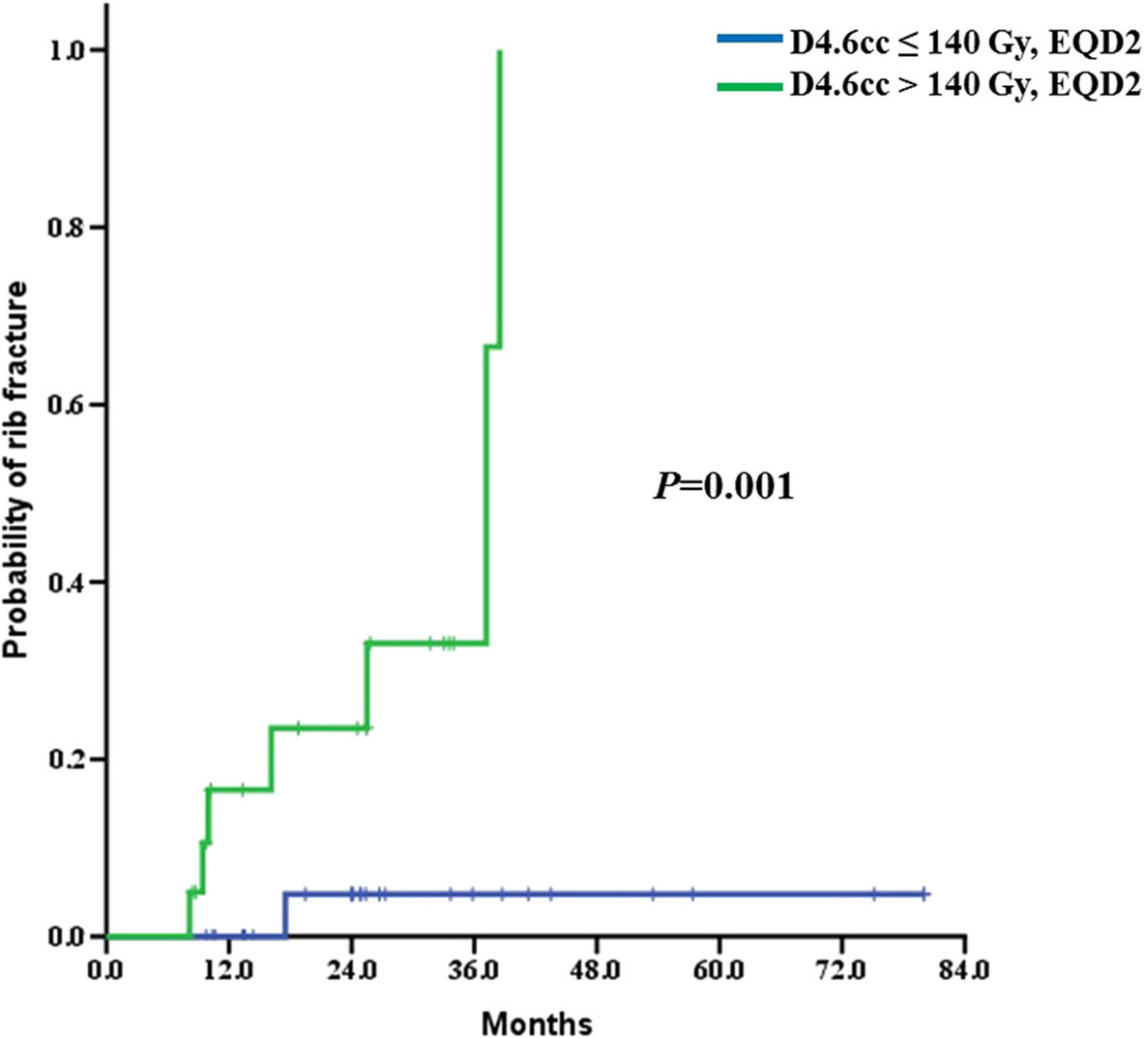
Cumulative incidence of radiation induced rib fracture after SABR

A representative case of dose distribution and rib fracture on CT image is shown in Fig. 2. The results of univariate and multivariate analysis are summarized in Table 2. On univariate analysis, no clinical factors showed significant association with RIRF but rib-tumor distance, D_4.6cc_ and V_160_ were significant risk factors (*p =* 0.035, *p =* 0.001 and *p =* 0.000, respectively). Three factors, rib-tumor distance, D_4.6cc_ and V_160_ were significantly associated to each other, therefore, to avoid multicollinearity, only D_4.6cc_ was included on multivariate analysis. On multivariate analysis, D_4.6cc_ was confirmed as a significant risk factor for RIRF (*p =* 0.009). Cumulative incidence of RIRF according to the D_4.6cc_ is shown in Fig. 3.

**Table 2:** Univariate and multivariate analysis of risk factors for radiation induced rib fracture

		Rib fracture		*p-*value	
Variables		No	Yes	Univariate	Multivariate
Sex	Male	26	4	0.493	NS
	Female	15	4		
Age	≤65	18	3	0.636	NS
	>65	23	5		
Tumor location	Upper lobe	15	4	0.086	NS
	Lower lobe^a^	26	4		
Multiple treatment	No	24	5	0.759	NS
	Yes	17	3		
DM	No	32	6	0.581	NS
	Yes	9	2		
COPD	No	35	5	0.061	NS
	Yes	6	3		
GTV volume	≤17 cc	33	4	0.097	NS
	>17 cc	8	4		
Rib-tumor distance	≤0.4 cm	21	8	0.035	_
	>0.4 cm	20	0		
D_4.6cc_	≤140 Gy, EQD2	27	1	0.001	0.009
	>140 Gy, EQD2	21	7		
V_160_	≤3.2 cc	27	0	0.000	_
	>3.2 cc	22	8		

^a^includes middle lobe in case of right lung

*DM* diabetes mellitus, *COPD* chronic obstructive pulmonary disease, *GTV* gross tumor volume

### 4D dose calculation

**Fig. 4:**
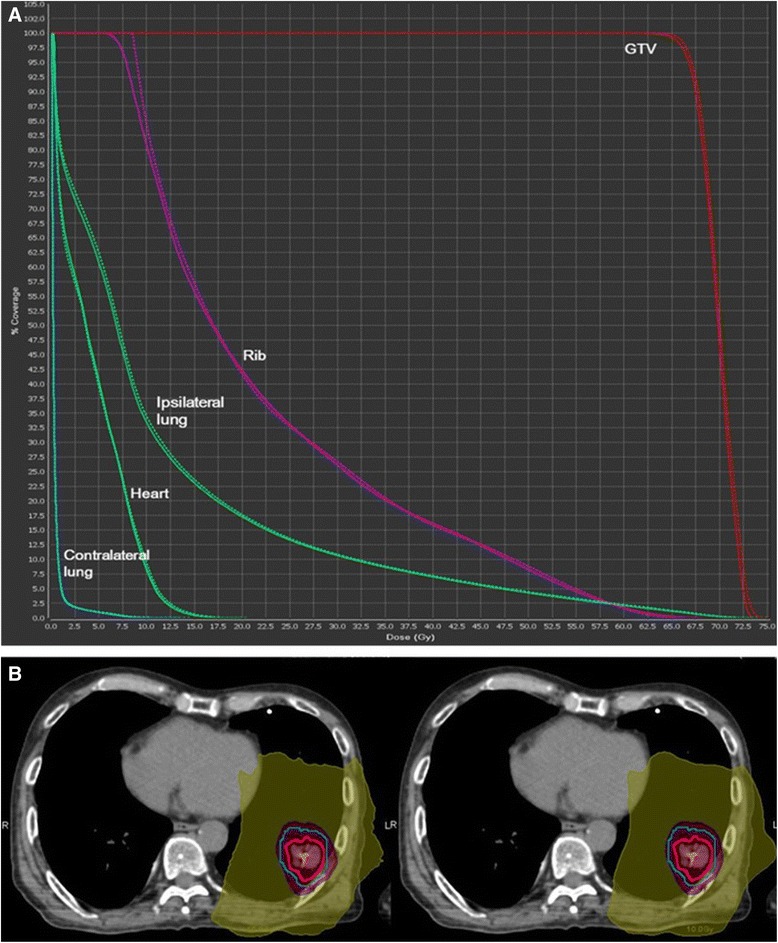
Comparison of 3D and 4D dose calculations in a representative patient. The male patient was treated for squamous carcinoma of *left lower lung* with 60 Gy in 3 fractions. The maximum diameter of tumor was 2.1 cm and the PTV was abutting the rib. After 1 year follow-up, no rib fracture was found. **a** DVH of two dose calculations. *Solid line*: 4D dose calculation, *Dashed line*: 3D calculation. **b** Representative transverse plane of dose distributions. Left: 3D calculation, right: 4D calculation

When the DVHs of 3D and 4D dose calculations were compared, no significant differences in dose to ribs (V_160_ and D_4.6cc_, *p* = 0.368 and *p* = 0.254, respectively by paired t-test) were found (Fig. 4). Tumor location, distance between rib and tumor, and tumor size did not affect the differences of two dose calculations. Difference between two dose calculations did not affect the development of rib fractures.

## Discussion

Overcoming the treatment errors caused by respiratory motion is a great challenge in radiotherapy for lung tumors. Various techniques have been suggested as solutions for this problem and real-time tumor tracking is one of the options [20]. In our study, the cumulative incidence of RIRF was 12.2 and 16.6% in 2 and 3 years, respectively. According to the previous studies, the incidence of RIRF after SABR varies from 20 to 40% [21–24]. Difference of selection criteria of included patients and different fractionation scheme precludes direct comparison between studies, however, the incidence of RIRF in our study was lower than those in previous studies, in general. Recently, Lischalk et al. reported long-term outcome of non-small cell lung cancer patients treated with Cyberknife® using fiducial tracking [25]. Although their incidence of rib fracture was higher (21.4% in 2 years) than that in our study, it was relatively lower than those in most other studies. Highly conformal dose distribution of Cyberknife® and real-time tumor tracking system could result in the low incidence of RIRF. Association between radiation dose to ribs and risk of rib fractures has been suggested in previous studies. Various dose-volume parameters such as D_max_, D_0.5cc_, D_8.0cc_ and V_30_ to V_70_ have been shown to be significant risk factors for RIRFs [21, 22, 24, 26–28]. In a pooled analysis of patients undergoing SABR with 2 different types of treatment machine (LINAC and Cyberknife®), 10% risk level for grade 2 or higher chest wall complication for D_2cc_ was 43.0 Gy in 4 fractions [29]. A recent study by Okoukoni et al. reported early cortical thinning of ribs after SABR in regions of receiving ≥10 Gy and suggested that rib thinning would contribute to the occurrence of rib fractures [30]. In our study, various parameters were associated with RIRF but D_4.6cc_ and V_160_ were most predictive. D_4.6cc_ less than EQD2 of 140 Gy, is equivalent to 41.7 Gy in 3 fractions which is higher than previously suggested cut-off values, D_max_ of 42.4 Gy in 4 fraction [21] or 54 Gy in 7–9 fractions [24] and D_8.0cc_ of 54 Gy in 4 fractions [26].

Rib-tumor distance was significantly associated with rib fractures in previous studies [18, 28]. Asai et al. suggested rib-tumor distance less than 2 cm as a significant risk factor [21] and Bongers et al. reported more grade 3 chest wall toxicity in tumors within 25 mm from chest wall [18]. But in our study, most of the lesions were located less than 2 cm from ribs (83.7%) and 0.4 cm was selected as cutoff value. Lesions close to chest wall inevitably result in higher dose to larger volume of ribs, therefore the rib-tumor distance was significantly associated with D_4.6cc_ and V_160_ (*P* < 0.05). Although rib-tumor distance was excluded in the multivariate analysis to exclude multicollinearity, it is suggested as a good predictor for RIRF.

Regarding tumor size, median gross tumor volume was 5.90 cc ranging from 0.79 cc to 111.74 cc, indicating that our study included larger lesions compared with other studies [18, 24, 27]. Larger tumor predicts larger dose to adjacent normal tissues and more toxicity is expected. Bongers et al. identified the PTV size as a significant risk factor for chest wall pain [18] and tumor dimension and PTV volume were correlated with chest wall toxicity in study by Stephans et al. [27].

As we have seen above, despite of unfavorable factors, our result showed low incidence of RIRF. All the patients in our study were treated with real-time tumor tracking system. The tumor tracking system has been shown to improve the treatment accuracy and low toxicity of SABR with real-time tumor tracking in lung cancer patients has been reported, although all these were single arm studies with no control groups [13, 15, 31]. Therefore, use of real-time tumor tracking in all patients should have contributed to the low incidence of RIRF.

Of the three patients with chest wall pain, one complained of chest wall pain at 2 months after SABR and the other two at 3 months after SABR. All these patients developed rib fractures with longer follow-up. Compatible with our result, reported duration from SABR to chest wall pain were generally shorter than the duration to rib fractures [7]. The mechanism of chest wall pain is thought to be different from that of rib fractures and the intercostal nerve injury is suggested as a possible cause of chest wall pain [32]. Because of steep dose gradient, irradiated volume of chest wall is small in our study and this might have contributed to the low incidence of chest wall pain. Although the relationship between chest wall pain and rib fractures is not identified, our results suggest the need for close observation of the patients with chest wall pain.

Because of respiration, lung undergoes continuous motion and deformation. But in clinical setting, 3D dose calculations are used for treatment planning for radiotherapy. This raised concerns about the discrepancies in planned dose and actually delivered dose to target. Several studies investigating this issue found that there were small difference between 3D and 4D calculations and 3D dose calculations can provide good approximation of 4D dose calculations in clinical setting [33–35]. Recent study by Chan et al. [36] argued that 4D Monte Carlo optimization provide more accurate planning than 3D optimization or 4D dose renormalization. However, according to their results, only conformity index was significantly different and the absolute differences were very small. Due to the small differences, the tumor control probability did not changed after 4D dose calculations. Moreover, there were no significant differences in dose to normal organs and normal tissue complication probability. It is remarkable that they showed the actual difference of 3D and 4D dose calculations but the differences have little impact on the clinical results and this support the results of our study. Most of the lesions included in our study were peripheral lesions with the median rib-tumor distances of 0.25 cm. Therefore, we expected 4D calculations could provide more accurate risk estimation for rib fractures, but no significant differences were found between 3D and 4D dose calculations. Tumor characteristics such as rib-tumor distances and tumor locations did not affect the difference of two dose calculations. Therefore, our results confirmed the result of previous studies and 3D dose calculation can be used to predict the normal tissue toxicity in SABR for lung tumors. Because of limited number of lesions included in our study, further studies involving more patients in prospective setting are needed to validate this.

There are several limitations to our study. First, it is retrospective study and the interval of follow-up evaluations and image work up was not exactly the same in all the patients. Therefore patient reported outcomes such as low grade chest wall pain might be underestimated and the development of asymptomatic rib fractures could be recorded with a delay. Second, included patients were treated with various fractionation scheme and some patients received multiple course of thoracic radiotherapy. Although, we used linear-quadratic model and calculated EQD2 and DVH parameters were obtained from summated dose of multiple treatment plans, this heterogeneity of study population might have caused undetected bias or errors. However, in clinical settings, more diverse fractionation schemes are used and more patients are undergoing multiple course of radiotherapy, inclusion of various fractionation schemes and heterogeneous patients could provide more practical guideline.

## Conclusions

In conclusion, SABR in primary or metastatic lung tumors using real-time tumor tracking provides excellent tumor controls with low incidence of chest wall toxicity. Rib-tumor distance, D_4.6cc_ and V_160_ are significant risk factor for RIRF and by limiting dose to the 4.6 cc volume of rib would prevent RIRF. In clinical setting, 3D dose calculations can substitute 4D dose calculations for estimation of rib fracture risks. This results need to be validated in the future studies.

## Abbreviations

3D: 3-dimensional
4D: 4-dimensional
AUC: Areas under the curves
CT: Computed tomography
D_max_: Maximum point dose of irradiated rib
D_V_: Absolute dose received by rib volumes of V
DVH: Dose-volume histogram
EQD2: 2 Gy equivalent dose
GTV: Gross tumor volume
PTV: Planning target volume
RIRF: Radiation induced rib fracture
SABR: Stereotactic ablative body radiotherapy
V_D_: Absolute rib volume receiving a threshold dose D

## References

[1] Nagata Y, Takayama K, Matsuo Y, Norihisa Y, Mizowaki T, Sakamoto T, Sakamoto M, Mitsumori M, Shibuya K, Araki N (2005). Clinical outcomes of a phase I/II study of 48 Gy of stereotactic body radiotherapy in 4 fractions for primary lung cancer using a stereotactic body frame. Int J Radiat Oncol Biol Phys.

[2] Timmerman R, Paulus R, Galvin J, Michalski J, Straube W, Bradley J, Fakiris A, Bezjak A, Videtic G, Johnstone D (2010). Stereotactic body radiation therapy for inoperable early stage lung cancer. JAMA.

[3] Onishi H, Shirato H, Nagata Y, Hiraoka M, Fujino M, Gomi K, Karasawa K, Hayakawa K, Niibe Y, Takai Y (2011). Stereotactic body radiotherapy (SBRT) for operable stage I non-small-cell lung cancer: can SBRT be comparable to surgery?. Int J Radiat Oncol Biol Phys.

[4] Soldà F, Lodge M, Ashley S, Whitington A, Goldstraw P, Brada M (2013). Stereotactic radiotherapy (SABR) for the treatment of primary non-small cell lung cancer; Systematic review and comparison with a surgical cohort. Radiother Oncol.

[5] Verstegen N, Oosterhuis J, Palma D, Rodrigues G, Lagerwaard F, van der Elst A, Mollema R, van Tets W, Warner A, Joosten J (2013). Stage I–II non-small-cell lung cancer treated using either stereotactic ablative radiotherapy (SABR) or lobectomy by video-assisted thoracoscopic surgery (VATS): outcomes of a propensity score-matched analysis. Ann Oncol.

[6] Chang JY, Senan S, Paul MA, Mehran RJ, Louie AV, Balter P, Groen HJ, McRae SE, Widder J, Feng L (2015). Stereotactic ablative radiotherapy versus lobectomy for operable stage I non-small-cell lung cancer: a pooled analysis of two randomised trials. Lancet Oncol.

[7] Thibault I, Chiang A, Erler D, Yeung L, Poon I, Kim A, Keller B, Lochray F, Jain S, Soliman H, Cheung P (2016). Predictors of Chest Wall Toxicity after Lung Stereotactic Ablative Radiotherapy. Clin Oncol.

[8] Kong FM, Ritter T, Quint DJ, Senan S, Gaspar LE, Komaki RU, Hurkmans CW, Timmerman R, Bezjak A, Bradley JD (2011). Consideration of dose limits for organs at risk of thoracic radiotherapy: atlas for lung, proximal bronchial tree, esophagus, spinal cord, ribs, and brachial plexus. Int J Radiat Oncol Biol Phys.

[9] Shaikh T, Turaka A (2014). Predictors and management of chest wall toxicity after lung stereotactic body radiotherapy. Cancer Treat Rev.

[10] Lo SS, Sahgal A, Chang EL, Mayr NA, Teh BS, Huang Z, Schefter TE, Yao M, Machtay M, Slotman BJ, Timmerman RD (2013). Serious complications associated with stereotactic ablative radiotherapy and strategies to mitigate the risk. Clin Oncol.

[11] Dieterich S, Gibbs IC (2011). The CyberKnife in clinical use: current roles, future expectations.

[12] Schweikard A, Glosser G, Bodduluri M, Murphy MJ, Adler JR (2000). Robotic motion compensation for respiratory movement during radiosurgery. Computer Aided Surgery.

[13] van der Voort van Zyp NC, Prévost J-B, Hoogeman MS, Praag J, van der Holt B, Levendag PC, van Klaveren RJ, Pattynama P, Nuyttens JJ: Stereotactic radiotherapy with real-time tumor tracking for non-small cell lung cancer: Clinical outcome. Radiother Oncol. 2009;91:296–300. 10.1016/j.radonc.2009.02.011 19297048

[14] Nuyttens JJ, van de Pol M (2012). The CyberKnife radiosurgery system for lung cancer. Expert Rev Med Devices.

[15] Nuyttens J, Prevost J-B, Praag J, Hoogeman M, Van Klaveren R, Levendag P, Pattynama P (2006). Lung tumor tracking during stereotactic radiotherapy treatment with the CyberKnife: Marker placement and early results. Acta Oncol.

[16] Roth T, Rankine L, Schreiber E, Das S, Mavroidis P (2016). SU-F-T-546: a radiobiological comparative study of robotic and LINAC-based lung SBRT. Med Phys.

[17] Overgaard M (1988). Spontaneous radiation-induced Rib fractures in breast cancer patients treated with postmastectomy irradiation—a clinical radiobiological analysis of the influence of fraction size and dose–response relationships on late bone damage. Acta Oncol.

[18] Bongers EM, Haasbeek CJ, Lagerwaard FJ, Slotman BJ, Senan S (2011). Incidence and risk factors for chest wall toxicity after risk-adapted stereotactic radiotherapy for early-stage lung cancer. J Thorac Oncol.

[19] Pettersson N, Nyman J, Johansson KA (2009). Radiation-induced rib fractures after hypofractionated stereotactic body radiation therapy of non-small cell lung cancer: a dose- and volume-response analysis. Radiother Oncol.

[20] Giraud P, Yorke E, Jiang S, Simon L, Rosenzweig K, Mageras G (2006). Reduction of organ motion effects in IMRT and conformal 3D radiation delivery by using gating and tracking techniques. Cancer/Radiothérapie.

[21] Asai K, Shioyama Y, Nakamura K, Sasaki T, Ohga S, Nonoshita T, Yoshitake T, Ohnishi K, Terashima K, Matsumoto K (2012). Radiation-induced rib fractures after hypofractionated stereotactic body radiation therapy: risk factors and dose-volume relationship. Int J Radiat Oncol Biol Phys.

[22] Taremi M, Hope A, Lindsay P, Dahele M, Fung S, Purdie TG, Jaffray D, Dawson L, Bezjak A (2012). Predictors of radiotherapy induced bone injury (RIBI) after stereotactic lung radiotherapy. Radiat Oncol.

[23] Nambu A, Onishi H, Aoki S, Tominaga L, Kuriyama K, Araya M, Saito R, Maehata Y, Komiyama T, Marino K (2013). Rib fracture after stereotactic radiotherapy for primary lung cancer: prevalence, degree of clinical symptoms, and risk factors. BMC Cancer.

[24] Aoki M, Sato M, Hirose K, Akimoto H, Kawaguchi H, Hatayama Y, Ono S, Takai Y (2015). Radiation-induced rib fracture after stereotactic body radiotherapy with a total dose of 54–56 Gy given in 9–7 fractions for patients with peripheral lung tumor: impact of maximum dose and fraction size. Radiat Oncol.

[25] Lischalk JW, Woo SM, Kataria S, Aghdam N, Paydar I, Repka MC, Anderson ED, Collins BT (2016). Long-term outcomes of stereotactic body radiation therapy (SBRT) with fiducial tracking for inoperable stage I non-small cell lung cancer (NSCLC). J Radiat Oncol.

[26] Kim SS, Song SY, Kwak J, Ahn SD, Kim JH, Lee JS, Kim WS, Kim S-W, Choi EK (2013). Clinical prognostic factors and grading system for rib fracture following stereotactic body radiation therapy (SBRT) in patients with peripheral lung tumors. Lung Cancer.

[27] Stephans KL, Djemil T, Tendulkar RD, Robinson CG, Reddy CA, Videtic GM (2012). Prediction of chest wall toxicity from lung stereotactic body radiotherapy (SBRT). Int J Radiat Oncol Biol Phys.

[28] Dunlap NE, Cai J, Biedermann GB, Yang W, Benedict SH, Sheng K, Schefter TE, Kavanagh BD, Larner JM (2010). Chest wall volume receiving >30 Gy predicts risk of severe pain and/or rib fracture after lung stereotactic body radiotherapy. Int J Radiat Oncol Biol Phys.

[29] Kimsey F, McKay J, Gefter J, Milano MT, Moiseenko V, Grimm J, Berg R (2016). Dose–response model for chest wall tolerance of stereotactic body radiation therapy. Semin Radiat Oncol.

[30] Okoukoni C, Lynch SK, McTyre ER, Randolph DM, Weaver AA, Blackstock AW, Lally BE, Munley MT, Willey JS (2016). A cortical thickness and radiation dose mapping approach identifies early thinning of ribs after stereotactic body radiation therapy. Radiother Oncol.

[31] Inoue T, Katoh N, Onimaru R, Shimizu S, Tsuchiya K, Suzuki R, Sakakibara-Konishi J, Shinagawa N, Oizumi S, Shirato H (2013). Stereotactic body radiotherapy using gated radiotherapy with real-time tumor-tracking for stage I non-small cell lung cancer. Radiat Oncol.

[32] Andolino DL, Forquer JA, Henderson MA, Barriger RB, Shapiro RH, Brabham JG, Johnstone PA, Cardenes HR, Fakiris AJ (2011). Chest wall toxicity after stereotactic body radiotherapy for malignant lesions of the lung and liver. Int J Radiat Oncol Biol Phys.

[33] Rosu M, Balter JM, Chetty IJ, Kessler ML, McShan DL, Balter P, Ten Haken RK (2007). How extensive of a 4D dataset is needed to estimate cumulative dose distribution plan evaluation metrics in conformal lung therapy?. Med Phys.

[34] Rao M, Wu J, Cao D, Wong T, Mehta V, Shepard D, Ye J (2012). Dosimetric impact of breathing motion in lung stereotactic body radiotherapy treatment using intensity modulated radiotherapy and volumetric modulated arc therapy [corrected]. Int J Radiat Oncol Biol Phys.

[35] Chan MK, Kwong DL, Ng SC, Tong AS, Tam EK (2013). Experimental evaluations of the accuracy of 3D and 4D planning in robotic tracking stereotactic body radiotherapy for lung cancers. Med Phys.

[36] Chan MK, Werner R, Ayadi M, Blanck O (2015). Comparison of 3D and 4D Monte Carlo optimization in robotic tracking stereotactic body radiotherapy of lung cancer. Strahlenther Onkol.

