# Erratum to: Predictors of chest wall toxicity after stereotactic ablative radiotherapy using real-time tumor tracking for lung tumors

**DOI:** 10.1186/s13014-017-0857-1

**Published:** 2017-07-21

**Authors:** Younghee Park, Hee Jung Kim, Ah Ram Chang

**Affiliations:** Department of Radiation Oncology/CyberKnife Center, Soonchunhyang University Seoul Hospital, 59 Daesagwan-ro, Yongsan-gu, Seoul, 140-743 Republic of Korea

## Erratum

Upon publication of the original article [1], it was noticed that the Funding Section was missing. The Funding section should read as “This work was supported by the Soonchunhyang University Research Fund”. This has now been acknowledged and corrected in this erratum.
